# Moderate Aerobic Exercise Reduces the Detrimental Effects of Hypoxia on Cardiac Autonomic Control in Healthy Volunteers

**DOI:** 10.3390/jpm13040585

**Published:** 2023-03-27

**Authors:** Luca Giovanelli, Mara Malacarne, Massimo Pagani, Gianni Biolo, Igor B. Mekjavic, Giuseppina Bernardelli, Daniela Lucini

**Affiliations:** 1 BIOMETRA Department, University of Milan, 20129 Milan, Italy; 2 Exercise Medicine Unit, Istituto Auxologico Italiano, IRCCS, 20135 Milan, Italy; 3 Department of Medical Sciences, University of Trieste, 34149 Trieste, Italy; 4 Department of Automatics, Biocybernetics and Robotics, Jožef Stefan Institute, SI-1000 Ljubljana, Slovenia; 5 DISCCO Department, University of Milan, 20122 Milan, Italy

**Keywords:** physical activity, physical inactivity, aerobic exercise, bedrest, hypoxia, heart rate variability, autonomic nervous system, baroreflex sensitivity, vagal activity

## Abstract

Physical inactivity increases cardiometabolic risk through a variety of mechanisms, among which alterations of immunological, metabolic, and autonomic control systems may play a pivotal role. Physical inactivity is frequently associated with other factors that may further worsen prognosis. The association between physical inactivity and hypoxia is particularly interesting and characterizes several conditions—whether physiological (e.g., residing or trekking at high altitude and space flights) or pathological (e.g., chronic cardiopulmonary diseases and COVID-19). In this randomized intervention study, we investigated the combined effects of physical inactivity and hypoxia on autonomic control in eleven healthy and physically active male volunteers, both at baseline (ambulatory) conditions and, in a randomized order, hypoxic ambulatory, hypoxic bedrest, and normoxic bedrest (i.e., a simple experimental model of physical inactivity). Autoregressive spectral analysis of cardiovascular variabilities was employed to assess cardiac autonomic control. Notably, we found hypoxia to be associated with an impairment of cardiac autonomic control, especially when combined with bedrest. In particular, we observed an impairment of indices of baroreflex control, a reduction in the marker of prevalent vagal control to the SA node, and an increase in the marker of sympathetic control to vasculature.

## 1. Introduction

Regular aerobic physical activity reduces the risk and/or improves the prognosis of many chronic diseases such as coronary artery diseases, hypertension, cardiac failure, diabetes, obesity, and cancer [1,2]. According to recent data, adhering to a healthy lifestyle before infection with SARS-CoV-2 was associated with a lower risk of post-COVID-19 conditions [3], while physical inactivity was associated with a higher risk of a severe COVID-19 outcome [4]. The increased cardiometabolic risk linked to physical inactivity is mediated through a variety of mechanisms, among which alterations of immunological, metabolic, and autonomic control systems may play a pivotal role [5,6,7,8]. Physical inactivity can often be associated with other conditions that may further worsen the prognosis. Of particular interest is the association between physical inactivity and hypoxia. Such a condition is often present in real life, characterizing, for instance, patients affected by chronic cardiopulmonary pathologies, such as COPD, pulmonary fibrosis, obstructive sleep apnea (OSA), or heart failure. Moreover, hypoxia may be observed in particular scenarios, such as residing/trekking at high altitude or during space flights. A paper by Biolo et al. [9] showed that a number of pro- and anti-atherogenic pathways were activated following 10-day hypoxia and that this was potentiated by bedrest (a simple experimental model of physical inactivity). On the other hand, a moderate level of physical activity under hypoxia conditions was observed to enhance anti-atherogenic pathways and lower cardiometabolic risk.

Alterations in cardiac autonomic regulation (CAR) may represent a further important mechanism to consider. Autonomic nervous system (ANS) control is in fact impaired in diabetes, obesity, hypertension, and coronary artery disease, with a dominant activity of the sympathetic system over the vagal one [10,11,12,13,14], which ultimately results in structural and functional abnormalities in the cardiovascular system [15]. Notably, aerobic endurance exercise has been shown to significantly ameliorate autonomic balance, with consequent benefits in preventing and treating multiple chronic non-communicable diseases [1,16,17,18,19,20,21]. Even patients suffering from OSA, as well as astronauts after space flights, present an increased cardiometabolic risk [22] and impaired CAR, [23,24,25]. In fact, between hypoxia and CAR, there seems to be a complex bidirectional interaction featuring closed-loop circuits from the periphery to the central autonomic network [26]. In particular, hypoxia accelerates heart rate (HR) by increasing sympathetic activity and decreasing vagal tone in order to maintain systemic oxygen delivery [27]. While sympathetic activation is directly triggered by the arterial chemoreflex [28], the regulation of vagal withdrawal appears more intricate. In contrast to anaesthetized dogs, where the hypoxia-induced vagal withdrawal was shown to be governed by a pulmonary inflation reflex [29,30], in humans, vagal withdrawal occurs in response to chemoreflex activation regardless of any increase in pulmonary ventilation [31].

In the present study, we investigated the combined effects of physical inactivity and hypoxia on autonomic control in the same cohort enrolled by Biolo and coauthors [9]. Healthy volunteers were studied at baseline (ambulatory) conditions and, in a randomized order, hypoxic ambulatory, hypoxic bedrest, and normoxic bedrest. Intriguingly, our findings might have a prominent translational relevance; that is, corroborating the use of aerobic exercise as a preventive/therapeutic tool to minimize the detrimental effects on CAR in hypoxic conditions—whether physiological (e.g., subjects residing or trekking at high altitude and astronauts) or pathological (e.g., patients affected with chronic cardiopulmonary pathologies).

## 2. Materials and Methods

### 2.1. Study Design

The present study is based on the analysis of the heart rate variability (HRV) data obtained from the project ‘LunHab: Lunar Habitat Simulation’ [32], which took place at the Olympic Sport Centre Planica (Ratece, Slovenia). The protocol conformed to the Declaration of Helsinki and the following amendments, and was approved by the National Committee for Medical Ethics at the Ministry of Health of the Republic of Slovenia (205/2/11). Written informed consent was obtained from each participant [32]. The detailed protocol has already been published in previous papers [9,32]. In brief, eleven healthy and physically active male volunteers (age 24 ± 4 years; body mass index 22 ± 2 kg·m^−2^) completed the following experimental campaigns in a randomized order: (a) normoxic bedrest (inspired partial pressure of oxygen: 21 kPa); (b) hypoxic bedrest (inspired partial pressure of oxygen: about 12.5 kPa); and (c) hypoxic ambulatory. Before the intervention, subjects were evaluated in normoxic ambulatory conditions (baseline) mimicking the respective levels of daily activity. Each campaign lasted 20 days (5 days: dietary and environmental adaptation; 10 days: intervention; 5 days: recovery) and was spaced from the others by a 1-month wash-out period. Across the study periods, five meals per day (three main meals and two snacks) were provided to maintain the eucaloric condition. Normobaric hypoxia was achieved in a hypoxic facility simulating 4000 m of altitude. During the hypoxic ambulatory confinement, participants were asked to perform, in addition to their daily activity, two moderate intensity exercise sessions per day (comprising 30 min of stepping on a 30 cm step), with an individually targeted HR corresponding to approximately 60% of maximal HR. This timing allowed us to ensure that, during ambulatory conditions, subjects were active to a similar extent, both without and with hypoxic stimulus, thus avoiding an activity bias. Likewise, bedrest condition (i.e., a simple experimental model of physical inactivity) was the same without and with hypoxic stimulus.

### 2.2. Cardiac Autonomic Control

On the last day of every session, participants underwent a non-invasive evaluation of their autonomic nervous system before breakfast in the morning. Following the placement of the electrodes, respiration transducer (piezoelectric sensor), and plethysmographic finger cuff for arterial pressure measurement (Finometer, NL), a continuous recording was carried out for 10 min. Data were acquired at 500 samples/s on a PC and stored on a hard disk. Data analysis was then performed using ad hoc software (Heartscope) that implements the autoregressive spectral analysis algorithms based on the sympatho-vagal model [26,33,34]. All data were artefact-free and the analyzed tachogram contained only sinus beats. Technically acceptable autospectra of the RR interval usually contain three major components, centred at about 0, 0.10, and 0.25 Hz. In addition, some small (<5%) noise components may also be present and disregarded. Low- (L) and high-frequency (H) components of RR interval variability (V) are presented in both absolute (msec^2^) and normalized units (nu). The HF component, obtained from spontaneous breathing, is synchronous with the major respiratory component, having an elevated coherence (above 0.5). Occasionally, spontaneous breathing inches below 0.12 Hz and the LF and HF components merge into a single major frequency (around 0.1 Hz), thus representing an entrainment that leads to discarding uninterpretable data. Using a bivariate approach (RR V and AP V), if the coherence of simultaneous LF and HF components of the AR spectra of the RR tachogram and of arterial pressure V is sufficiently high (>0.5), the following formula gives the value (msec·mmHg^−1^) of the frequency domain α index mean (AlphaM), estimating the cardiac baroreflex gain: 0.5 × √{[(LF^2^ RR)/(LF^2^ SAP)] + [(HF^2^ RR)/(HF^2^ SAP)]}/2 [35]. For the sake of completeness, we also reported a time-domain approach (baroreflex slope, BRS) [36]. Lastly, by means of a simple algebraic computation using a trivariate (RR, SAP, and respiration) model, we estimated the value of cardiopulmonary baroreflex (Alpha CP) [37].

### 2.3. Statistics

Data are presented as mean ± SD. Differences among the four experimental epochs (ambulatory at entry and the end of the hypoxia campaign, as well as bedrest alone or combined with hypoxia) were explored with a mixed-model approach considering these four conditions as fixed factors and assessing paired contrasts. The possible bias of non-normality was minimized using Conover’s rank transformation [38]. The IBM SPSS SW tool (IBM, Armonk, USA) versus 28 was employed for computations.

## 3. Results

Table 1 reports the definitions of the variables (proxies of autonomic control) employed in the study.

Table 2 reports the descriptive statistics of the main RR and SAP variability variables in the four considered campaigns. The first and second columns (ambulatory) refer to conditions (normoxia or hypoxia) in which subjects may perform physical activity, while the third and fourth columns (bedrest) refer to conditions (normoxia or hypoxia) characterized by physical inactivity.

**Figure 1:**
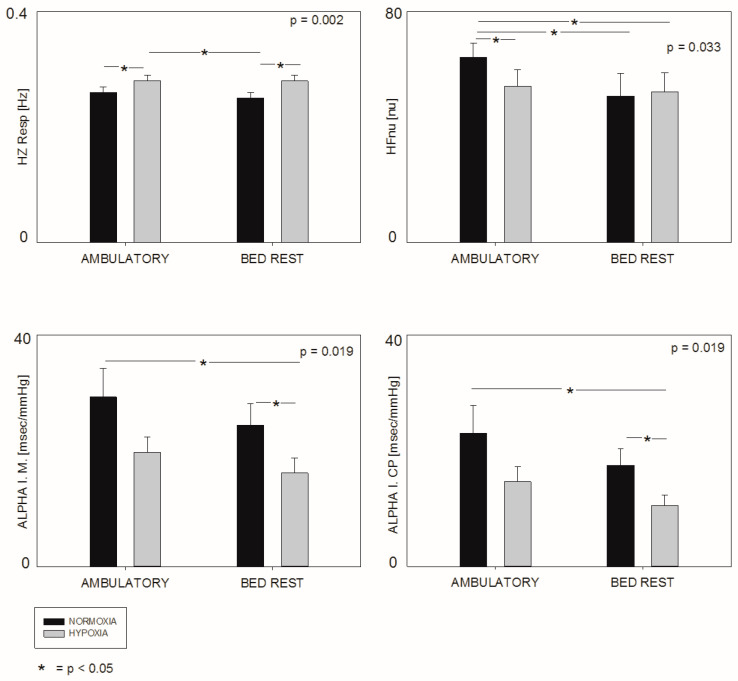
Significant differences in the cardiac autonomic regulation indexes among the four campaigns.

Data at baseline were all within the usual values for healthy subjects. Respiratory entrainment was never observed in participants. Respiratory frequency (see Figure 1) was significantly different among the four epochs (*p* = 0.002); in particular, it was increased in hypoxic campaigns, during both ambulatory (baseline) and bedrest conditions (characterized by physical inactivity).

RR HFnu (see Figure 1), a marker of prevalent vagal modulation to the SA node, was significantly different considering the four campaigns overall (*p* = 0.033). In particular, contrast differences showed that hypoxia was associated with a significant reduction in this marker in ambulatory campaigns, but not in bedrest conditions. Hypoxia combined with bedrest was associated with a further reduction in this marker as compared with normoxic ambulatory campaigns (baseline).

SAP LFa, a marker of prevalent sympathetic modulation to the vasculature, was significantly different in the four campaigns (*p* = 0.042), being higher during hypoxic stimuli. The Alpha Index M, a proxy of the overall gain of cardiac baroreflex sensitivity, was significantly different among the four campaigns (*p* = 0.019), being significantly reduced in hypoxic bedrest conditions as compared with ambulatory normoxia (see Figure 1). Similar significant differences were observed regarding the Alpha Index CP, a trivariate-model-derived proxy of the cardiopulmonary baroreflex. No significant differences were noted in heart rate, systolic arterial pressure, total variance, and LFRR nu (a marker of prevalent sympathetic modulation to the SA node). 

## 4. Discussion

In this randomized intervention study on eleven healthy and physically active male volunteers, we observed that hypoxia was associated with an impairment of cardiac autonomic control, especially when combined with bedrest (an experimental model of physical inactivity). In particular, we observed an impairment of the indices of baroreflex control, a reduction in the marker of prevalent vagal control to the SA node, and an increase in the marker of sympathetic control to the vasculature.

Bedrest represents a convenient model to study physical inactivity in healthy subjects. It was employed to study the effects of immobilization and/or lack of exercise characterizing particular conditions that are gaining momentum, such as space flight [24]. Bedrest studies are frequently performed in the head-down position, which increases the blood return to the cardiopulmonary circulation, likely amplifying the autonomic changes as a reflection of adding cardiopulmonary hemodynamics to inactivity. Notably, several studies found this condition to be associated with an impairment of the immune [39,40] and endocrine [41,42] systems as well as cardiac autonomic control [24,43,44,45]. Recently, Ferretti et al. [24] reported that, after prolonged head-down bedrest, RR and its variance, baroreceptor reflex sensitivity, and direct measures of tonic muscle sympathetic nerve activity were significantly reduced, while oscillatory indices were largely spared (a slight 15% reduction in RRHFnu was, however, observed late during bedrest). On the other hand, exercise was proposed as an important and relatively simple countermeasure to apply in order to minimize the detrimental effects of inactivity [36,39,46], even in extreme conditions such as in space [36]. In this paper, we confirm that, during normoxia, (simple) bedrest was associated with an impairment of cardiac autonomic control. In particular, we observed a significantly lower HFRRnu, a marker of vagal control of the SA node. Further, the indices’ proxies of baroreflex control seem to be reduced in the same conditions, even though time-domain indices appear less sensitive. 

In the present paper, we aimed to investigate the association between physical inactivity (obtained while maintaining subjects in the horizontal position—bedrest) and hypoxia, which characterizes several conditions—whether physiological (e.g., subjects residing or trekking at high altitude or simulation of microgravity) or pathological (e.g., patients affected by chronic cardiopulmonary pathologies such as chronic obstructive pulmonary disease, pulmonary fibrosis, heart failure, and OSA). Biolo and coworkers [9] showed, in the same subjects as in the present study, that a number of pro- and anti-atherogenic pathways were activated following hypoxia, with these alterations exacerbated by bedrest. On the other hand, a moderate level of physical activity in hypoxia conditions enhanced anti-atherogenic pathways and reduced cardiometabolic risk. In our paper, we found these metabolic changes to be accompanied by a similar impairment in autonomic cardiovascular control as well. In ambulatory (baseline) campaigns, hypoxia significantly reduced the marker of vagal control of the SA, while in bedrest campaigns, the detrimental effect of hypoxia was less evident, thus suggesting an important adjuvant effect of inactivity. This finding was corroborated by the results considering the indices of the overall baroreflex sensitivity (Alpha Mean and Alpha CP were clearly reduced during bedrest, particularly in hypoxic conditions) and the marker of sympathetic control of the vasculature (SAPLFa was overall higher during hypoxia and bedrest).

Regular physical exercise represents a pivotal strategy in the prevention and management of chronic diseases. Moreover, it is a tool to counterbalance the detrimental effects of inactivity in extreme conditions (such as space flights) that are characterized by possible hypobaric hypoxia as well [36]. In light of the beneficial effects of aerobic exercise in a hypoxic environment, hypoxic training is leveraged by competitive athletes to improve their performance in normoxia. For instance, a six-week aerobic continuous and interval training program under hypobaric hypoxia has been demonstrated to enhance both hemodynamic and autonomic function, with consequently better performance in amateur male swimmers [47]. Crucially, prescription of hypoxic exercise should be based on HR in order to ensure physiological cardiac autonomic responses that are similar to normoxic exercise, thus minimizing the impact of acute hypoxia on metabolic stress and cardiac autonomic recovery [48]. In our paper, we paid particular attention to maintaining an adequate endurance physical exercise dose in ambulatory hypoxia; that is, subjects had to perform a tailored moderate endurance exercise protocol (following a training heart rate of 60% of maximum HR). Moderate-intensity endurance exercise is recommended by international guidelines [1] in order to reach an important clinical goal such as a reduction in overall mortality or prevention/management of cardiometabolic/oncologic diseases. Exercise is also recommended in OSA patients [49], being able to improve many mechanisms underlying this condition. In several randomized controlled studies on the relationship between OSA and aerobic exercise [50,51,52,53,54], physical exercise has been demonstrated to improve this condition without necessarily changing body mass index. Our results suggest a possible improvement in cardiac autonomic regulation, which is impaired in OSA patients [55,56]. Of particular interest in this historical period are the beneficial effects of regular exercise in patients with SARS-CoV-2 infection, frequently presenting with respiratory problems. Regular exercise was associated with a better prognosis and lower risk of post-COVID-19 complications [3]. Recent data show an autonomic dysfunction in long COVID-19 patients as well [57], thus suggesting regular exercise as a possible additional management strategy.

High-altitude exposure may represent a further condition in which a hypoxic stimulus may have important consequences on health, particularly in patients affected with cardiometabolic diseases or non-acclimatized patients [58,59,60]. Many of these include the cardiovascular physiological or pathophysiological adaptational changes induced by high-altitude exposure, and autonomic nervous system imbalance may also play a role in this context [58,59,60,61]. In this paper, we paid attention to inducing hypoxic stimulus; normobaric hypoxia was achieved in a hypoxic facility simulating 4000 m of altitude. The shift in the O_2_/N_2_ ratio was attained slowly in order to minimize discomfort and dyspnea. Data derived from our study may corroborate the observation that endurance exercise before being exposed to high altitude may represent a tool to minimize the risk of high-altitude illness and adverse cardiovascular events [60].

In addition to an impairment of cardiac baroreflex, our approach permitted us to observe a (trivariate-model-derived) cardiopulmonary gain impairment. Baroreflex performance is frequently estimated by simplified models that consider the RR interval to be driven solely by arterial pressure, disregarding the critical role of other factors, such as variability noise, respiration, and the cardiopulmonary component. Although simplified, the trivariate model suggests that hypoxia and bedrest (alone or combined) lead to a selective impairment of the cardiopulmonary baroreflex.

Several limitations should be considered. The first one is the small sample size, although study subjects were well selected and resided in an ad hoc facility. All of the campaigns were clearly separated from each other and randomized. Besides, autonomic effects were inferred from an indirect method implying the use of a simplified cardiorespiratory model. Such a model, although imperfect, considers the physiological subdivision in arterial, cardiac, and pulmonary components, previously employed in various circumstances. Nevertheless, the methodology used was non-invasive, thus avoiding the possible confounding effect induced by invasive techniques (such as muscle nerve sympathetic activity recordings) that may cause pain and stress. Lastly, our study considered only healthy male volunteers. We did not study female subjects or patients with pulmonary impairment.

## 5. Conclusions

We have shown that a few days of physical inactivity (as obtained with bedrest)—alone or combined with hypoxia—impair autonomic cardiac regulation, as described by a simplified trivariate model, with the combination being more effective. In light of this observation and the changing point of view, we might conclude that the presence of moderate aerobic exercise (as observed during the hypoxic campaign while maintaining moderate exercise) reduces the detrimental effects of hypoxia on cardiac autonomic control in healthy volunteers. Notably, our findings might have a prominent translational relevance, corroborating the use of aerobic exercise as a preventive/therapeutic tool to minimize the detrimental effects on CAR in hypoxic conditions—whether physiological (e.g., subjects residing or trekking at high altitude and astronauts) or pathological (e.g., patients affected by chronic cardiopulmonary pathologies such as chronic obstructive pulmonary disease, pulmonary fibrosis, heart failure, and OSA).

## Figures and Tables

**Table 1:** Definitions of the variables (ANS proxies) employed in the study.

Variables	Units	Definition
HR	beat/min	Heart rate
RR Mean	msec	Average of RR interval from tachogram
RR V	msec^2^	RR variance (total power) from tachogram (proxy of vagal drive)
RR LFa	msec^2^	Absolute power (a) of low-frequency (LF) component of RR variability (V)
RR HFa	msec^2^	Absolute power (a) of high-frequency (HF) component of RRV
RR LFnu	nu	Normalized power (nu) of low-frequency (LF) component of RRV
RR HFnu	nu	Normalized power (nu) of high-frequency (HF) component of RRV
RR LF/HF	-	Ratio between absolute values of LF and HF
RR LFHz	Hz	Center frequency of RR LF
RR HFHz	Hz	Center frequency of RR HF, providing a measure of respiratory rate
Resp	Hz	Center frequency of respiration (piezoelectric sensor) spectra
SAP Mean	mmHg	Average of systogram (i.e., systolic arterial pressure variability by Finometer)
SAP V	mmHg^2^	Systolic arterial pressure variance from systogram
SAP LFa	mmHg^2^	Absolute power of LF component of systogram (proxy of vascular sympathetic drive)
SAP HFa	mmHg^2^	Absolute power of HF component of systogram
Alpha Index M	msec/mmHg	Frequency-domain (proxy) measure of cardiac baroreflex gain (proxy of vagal drive)
BRS	msec/mmHg	Time-domain measure of cardiac baroreflex slope (proxy of vagal drive)
A.XAR	msec/mmHg	Bivariate causal index of RR–SAP relationship
A.XXAR	msec/mmHg	Causal index from trivariate model of RR–SAP (avoiding respiration bias) relationship (proxy of arterial baroreflex)
Alpha Index CP	msec/mmHg	Trivariate model derived proxy of cardiopulmonary baroreflex

(a) Modified from Lucini et al., 2018 [35].

**Table 2:** Descriptive statistics of the main RR and SAP variability variables in the four considered campaigns.

Variables	Ambulatory		Bedrest		Cond	Contrast
	Normoxia	Hypoxia	Normoxia	Hypoxia		
HR (b/min)	61.52 ± 6.49	62.44 ± 5.92	61.62 ± 4.91	67.76 ± 7.95	0.111	
RR (ms)	985.24 ± 103.04	968.57 ± 89.17	979.15 ± 76.26	896.25 ± 100.67	0.111	
RR V (ms^2^)	4248.66 ± 4295.91	3406.52 ± 1701.65	3203.75 ± 1687.2	2468.54 ± 1416.04	0.192	
RR LFa (ms^2^)	1074.59 ± 987.12	1066.7 ± 544.24	957.6 ± 788.91	622 ± 625.06	0.100	
RR HFa (ms^2^)	1249.4 ± 1711.39	1025.39 ± 1122.01	888.24 ± 842.7	388.16 ± 313.8	0.063	
RR LFnu (nu)	47.96 ± 13.52	55.58 ± 14.59	53.48 ± 25.7	54.32 ± 17.59	0.168	
RR HFnu (nu)	48.14 ± 12.44	40.59 ± 14.63	38.04 ± 19.55	39.1 ± 16.62	**0.033**	**a b c**
LF/HF nu (au)	1.12 ± 0.55	1.61 ± 0.74	2.24 ± 2.08	1.68 ± 0.82	0.065	
RR LFHz (Hz)	0.1 ± 0.03	0.09 ± 0.03	0.1 ± 0.02	0.1 ± 0.03	0.957	
RR HFHz (Hz)	0.26 ± 0.05	0.28 ± 0.05	0.25 ± 0.06	0.27 ± 0.04	**0.009**	**a d**
RESP (Hz)	0.26 ± 0.05	0.28 ± 0.06	0.25 ± 0.06	0.28 ± 0.05	**0.002**	**a d f**
SAP (mmHg)	117.55 ± 12.35	117.24 ± 9.12	113.31 ± 17.21	121.21 ± 15.2	0.471	
SAP V (mmHg^2^)	24.48 ± 21.35	26.33 ± 17.93	23.90 ± 22.77	19.83 ± 16.04	0.447	
SAP LFa (mmHg^2^)	5.50 ± 4.02	13.44 ± 12.63	6.41 ± 10.45	6.93 ± 7.68	**0.042**	**d e**
SAP HFa (mmHg^2^)	1.22 ± 1.45	1.56 ± 1.93	1.36 ± 1.97	1.16 ± 1.01	0.053	
Alpha Index M (ms/mmHg)	29.30 ± 16.62	19.73 ± 9.06	24.38 ± 12.85	16.15 ± 8.63	**0.019**	**c f**
BRS (ms/mmHg)	28.08 ± 15.83	20.26 ± 4.28	25.99 ± 13.09	17.67 ± 8.48	0.089	
A.XAR (ms/mmHg)	7.56 ± 6.44	6.76 ± 3.70	8.27 ± 7.53	8.02 ± 6.42	0.662	
A.XXAR (ms/mmHg)	7.19 ± 6.48	5.05 ± 2.67	6.87 ± 7.02	5.58 ± 4.16	0.765	
Alpha Index CP (ms/mmHg)	23.00 ± 16.02	14.68 ± 8.84	17.51 ± 9.24	10.57 ± 6.05	**0.049**	**c f**

Data are presented as mean ± SD. Significant differences among campaigns (Cond) are provided by mixed-model repeated measures following Conover approximation. Variables with significant differences are indicated in bold. Abbreviations: HR: heart rate; RR: interbeat interval; V: variance, total power; LF: low frequency; HF: high frequency; Hz: Hertz; Resp: respiration; SAP: systolic arterial pressure; LFa: power of low-frequency component of SAP variability in ms^2^; Alpha Index M = frequency-domain index of cardiac baroreflex; BRS: baroreflex slope; A.XAR: bivariate-model-based causal index of cardiac baroreflex; A.XXAR: causal index of trivariate cardiac baroreflex; Alpha Index CP: cardiopulmonary baroreflex. Significant contrasts: **a** = ambulatory normoxia vs. ambulatory hypoxia, **b** = ambulatory normoxia vs. bedrest normoxia, **c** = ambulatory normoxia vs. bedrest hypoxia, **d** = ambulatory hypoxia vs. bedrest normoxia, **e** = ambulatory hypoxia vs. bedrest hypoxia, **f** = bedrest normoxia vs. bedrest hypoxia.

## Data Availability

Data is available on Zenodo (https://zenodo.org/badge/DOI/10.5281/zenodo.7774689.svg).
